# Secondary hormonal alterations in short-term severe hypothyroidism; in the focus: Apelin and copeptin

**DOI:** 10.3389/fendo.2022.981891

**Published:** 2022-09-14

**Authors:** Marin Gergics, Gréta Pham-Dobor, Zoltán Horváth-Szalai, Tamás Kőszegi, Emese Mezősi, László Bajnok

**Affiliations:** ^1^ 1st Department of Medicine, University of Pécs, Medical School, Pécs, Hungary; ^2^ Department of Laboratory Medicine, University of Pécs, Medical School, Pécs, Hungary; ^3^ János Szentágothai Research Centre, University of Pécs, Pécs, Hungary

**Keywords:** TSH, DTC (differentiated thyroid cancer), apelin, copeptin, hypothyroidism

## Abstract

**Objective:**

This study aimed to investigate the complex interactions of thyroid hormone, apelin, and copeptin in the fluid–ion homeostasis of patients with severe transitory hypothyroidism.

**Methods:**

In this prospective observational study, 39 patients (ECOG: 0; 11 men, 28 women, mean age: 50.3 ± 14.9 years) were investigated during short-term severe hypothyroidism due to surgical removal of the thyroid gland and after adequate thyroid replacement therapy. In addition to the routinely available lab tests, copeptin and apelin levels were determined using ELISA.

**Results:**

In the hypothyroid state, apelin concentration was lower, while copeptin levels did not differ compared to the euthyroid condition. Apelin showed a positive correlation with copeptin (*p* = 0.003), sodium (*p* = 0.002), NT-proBNP (*p* < 0.001), and fT4 (*p* < 0.001) and a negative correlation with thyroid-stimulating hormone (TSH) (*p* < 0.001). In multivariate linear regression models, copeptin and TSH proved to be significant independent predictors of apelin levels, of which TSH had an explanatory power of 48.7%. Aside from apelin, copeptin only correlated with sodium (*p* = 0.046). Sodium levels were negatively associated with TSH (*p* = 0.004) and positively with ACTH (*p* = 0.002) and cortisol (*p* = 0.047), in addition to copeptin. None of the parameters were independent predictors of serum sodium levels in a multivariate regression model.

**Conclusions:**

In short-term severe hypothyroidism, serum apelin level is markedly decreased, which may predispose susceptible patients to hyponatremia, while the level of copeptin is unchanged. TSH and copeptin are independent predictors of apelin concentration, of which TSH is stronger.

## Introduction

Hyponatremia, defined as plasma sodium concentration below 135 mmol/L, is the most common electrolyte disorder in hospitalized patients (1). Various conditions have been associated with hyponatremia, including chronic heart failure, chronic kidney disease, liver cirrhosis, hypovolemia, and the syndrome of inappropriate antidiuresis (SIAD), in which antidiuretic hormone (ADH)/arginine vasopressin (AVP) secretion occurs in the absence of an osmotic or hemodynamic abnormality (1, 2). Fluid–electrolyte imbalances, including impaired free water excretion, are common in hypothyroidism. Hyponatremia may evolve in this state; however, the exact mechanism and prevalence are not fully elucidated. ADH/AVP is released from the posterior pituitary due to increasing plasma osmolality and various stressors such as hypovolemia, hypoxia, acidosis, and severe infections (3). Hypovolemia is the main stimulus for ADH/AVP secretion, even in the presence of reduced serum osmolarity when hyponatremia may evolve. ADH/AVP acts on vasopressin-2 receptor (V2-R) in the collecting duct of the kidney to increase cAMP production and aquaporin-2 (AQP2) insertion into the apical membrane, leading to water reabsorption. Non-osmotic elevation of ADH/AVP has been related to cardiac fibrosis and dysfunction (4, 5). Some authors demonstrated an elevated level of ADH/AVP in hypothyroid patients that could be restored by thyroid hormone replacement (4, 6). In contrast, others found lower ADH/AVP in myxedematous patients (7), explaining hyponatremia with an ADH-independent mechanism of decreased water excretion. This could be the result of hypothyroidism-related kidney dysfunction with decreased glomerular filtration rate (GFR) and altered tubular activities (4, 8, 9). Methodological problems may partially explain these conflicting results since the measurement of ADH/AVP needs competitive assays due to the small size of ADH/AVP and is less reliable than the measurement of copeptin (10). Most of the ADH/AVP in the blood is bound to platelets (11), and prolonged storage of unprocessed blood samples may falsely elevate ADH/AVP levels (10, 11). Furthermore, ADH/AVP is unstable in aliquots when stored at −20°C or −80°C (12). Thus, routine measurement of circulating ADH/AVP levels could never be implemented in clinical patient care (10). Although copeptin is a more reliable humoral marker of ADH/AVP production (10), its alterations have not been investigated in hypothyroidism.

In addition to the elevation of copeptin levels, the altered secretion of further humoral factors may also play a role in the development of hyponatremia in SIAD. For example, the sex- and age-adjusted plasma levels for apelin and copeptin are 26% and 75% higher, respectively, in SIAD patients compared to healthy subjects (1, 13). The plasma apelin/copeptin ratio lies outside of the predicted range in 86% of SIAD patients. This finding emphasizes the primary osmoregulatory defect in these patients, where the hyponatremia is worsened by the inappropriately low plasma apelin concentration that cannot compensate for the increased ADH/AVP release (13).

Hypothalamic release of apelin is oppositely regulated by the plasma osmolality compared to ADH/AVP in animal and human models (14). Apelin is particularly abundant in the hypothalamic supraoptic and paraventricular nuclei, where it colocalizes with ADH/AVP in magnocellular neurons (15–18), blocking the ADH/AVP secretion. Apelin increases aqueous diuresis by increasing renal blood flow and by counteracting the antidiuretic effect of ADH/AVP on the distal convoluted and collecting tubules of the kidney (1, 19). Thus, apelin seems to play a key role in maintaining the fluid homeostasis. A lower apelin level may exert less blocking effects on the secretion and action of ADH/AVP.

In physiological conditions, ADH/AVP and apelin are released in balanced proportions from the magnocellular ADH/AVP neurons at levels appropriate for the current plasma osmolality (1). Following water deprivation, ADH/AVP is released from magnocellular vasopressinergic neurons into the bloodstream more rapidly than it is synthesized, causing the depletion of ADH/AVP in magnocellular neurons. In the meantime, the release of apelin decreases and apelin accumulates in magnocellular neurons. Thus, after dehydration, ADH/AVP and apelin are regulated in opposite manners, to facilitate systemic ADH/AVP release and suppress diuresis.

Following water loading, ADH/AVP release is decreased from magnocellular vasopressinergic neurons, causing an accumulation in ADH/AVP, while apelin release increases, leading to a depletion of apelin in magnocellular neurons. Thus, after water loading, ADH/AVP and apelin are regulated in opposite manners, to facilitate systemic apelin release and to increase aqueous diuresis.

The transcription of preproapelin, the precursor of the peptide hormone apelin, is present in other sites of the central nervous system, with the highest levels in the thalamus and frontal cortex (16, 20–22). Furthermore, apelin and/or its receptor can be found in tissues such as the placenta, heart, lung, kidney, liver, gastrointestinal tract, adrenals, uterus, ovaries (23–26), endothelial cells of various vessels, and plasma cells (27–29). Moreover, the expression of apelin increases during adipocyte differentiation (30). Apelin also plays a major role in blood pressure regulation as an endothelium-dependent vasodilator and has a positive inotropic effect by increasing cardiac output (19, 31). Hypoxia leads to the release of hypoxia-inducible factor (HIF-1) that upregulates the apelinergic signaling. However, it is not known how hypovolemia itself modulates the apelinergic system (1, 31).

Another humoral alteration demonstrated in hypothyroidism, paradoxically to the extracellular volume expansion, is the reduced level of atrial natriuretic hormone (ANH) (4, 32). Furthermore, patients with hypothyroidism often have lower plasma renin activity and plasma aldosterone concentrations (4) which can be explained as a humoral response to extracellular fluid retention.

Our study aimed to investigate the rather complex interactions of these humoral factors in the fluid–ion homeostasis in patients with severe transitory hypothyroidism.

## Materials and methods

### Patients

Patients between the age of 18 and 75 years with differentiated thyroid cancer (DTC) were included. Patients had no metastases and were scheduled for radioiodine (RAI) therapy after total or near-total thyroidectomy in this prospective, observational study. The patients were consecutively enrolled from 12 January 2018 to 21 February 2020.

They were in a complete endogenous or exogenous levothyroxine deficiency for at least 4 weeks before sampling. Hypothyroidism was confirmed in all patients by the determination of low serum free thyroxine (fT4), low serum free triiodothyronine (fT3), and markedly elevated thyroid-stimulating hormone (TSH). Patients were prospectively evaluated on the day of RAI therapy and on thyroxine 10–12 weeks later. DTC was not advanced (ECOG: 0). The exclusion criteria were any known chronic diseases or medications that could potentially interfere with thyroid status, including abnormal fluid retention due to heart, liver, or kidney diseases or diuretic treatment. Between the hypothyroid and control investigations, there were no i) changes in the medications beyond the thyroxine supplementation and ii) significant intercurrent diseases. During the 10–12 weeks follow-up, patients took their regular medications before control laboratory tests in the morning, including levothyroxine supplementation.

### Study protocol/design

Venous blood samples were obtained between 8 and 10 a.m. for further biochemical analysis. After that, body weight was measured to the nearest kilogram and height to the nearest centimeter. The waist circumference was gauged when the participant was standing upright with a tape measure in the horizontal plane midway between the lowest rib and the iliac crest, while the hip circumference was measured over the most pronounced area of the buttocks. The same investigator made all the anthropometric measurements to avoid interindividual variations.

Bioelectrical impedance analysis (BIA) (Bodystat Quadscan 4000, Bodystat Ltd., P.O. Box 50. IM99 1DQ Douglas, Isle of Man, United Kingdom) was used for the assessment of body composition. Patients were placed in the supine position for at least 5 min before the measurement. All participants had light clothing and their earrings, rings, bracelets, and any other metal were removed, which could influence the measurement results. Every measurement took approximately 30 s for each participant.

Only patients fulfilling the following BIA protocol were included: no alcohol consumption for 48 h, no strenuous activity for 12 h, and fasting for at least 4 h before the test. Patients with implanted electronic devices, like heart pacemakers, were excluded.

The study was carried out by the Declaration of Helsinki (2000) of the World Medical Association and approved by the Ethics Committee at the Medical Center of the University of Pécs (6961/2017). Subjects participated in the study after their written informed consent was obtained.

### Determination of routine laboratory tests and neurohormonal mediators

Venous blood samples were taken into plain tubes with accelerator gel and EDTA tubes (Vacutainer®, Becton Dickinson, Eysins, Vaud, Switzerland). Sera and plasma samples were obtained by centrifugation for 20 min at 2,300 rpm, then aliquoted in Eppendorf tubes, and stored at −80°C until analysis.

Chemistry panel, endocrine parameters, and fully automated blood picture tests were determined using the standard laboratory diagnostic kits and automated instrumentation of the Department of Laboratory Medicine, University of Pécs (accreditation number: NAH-1-1553/2016). The reference range for TSH was 0.27–4.2 mU/L, and for fT4, it was 12.0–22.0 pmol/L. Serum apelin and copeptin levels were measured with the ELISA method using Human Apelin ELISA kit (Catalog No. abx585113, Abbexa Ltd., UK, intra-assay CV <10%, inter-assay CV <10%), and Human Copeptin (CT-proAVP) ELISA kit (Catalog No. abx252269, Abbexa Ltd., UK, intra-assay CV <10%, inter-assay CV <10%) according to the manufacturers’ instructions on a BioTek Synergy HT plate reader at 450 nm. Serum N-terminal prohormone of brain natriuretic peptide (NT-proBNP) levels were measured using an immunoassay method using the Immulite 2000 automated instrument (Catalog No. L2KNT2, LNTCM, Siemens Healthcare Diagnostics Inc., USA).

### Statistical analysis

The Shapiro–Wilk test was used to check normal distributions on continuous variables. Data are presented as mean ± SD in parameters with normal distribution, while median and interquartile values are in the non-normal distribution. Comparisons of the two phases were made with paired sample *t*-test and Wilcoxon signed-rank test in normal and non-normal distributions, respectively. In the pairwise comparison, we applied the Bonferroni correction of the *p*-values to decrease the type I error. Correlation analysis calculating the Spearman coefficient was performed to assess the strength of the association between the different variables. Multiple regressions were used to determine the associations between clinical variables and serum concentrations of copeptin and apelin. The factors that were significantly associated with the single outcome at the univariate analysis and those considered to be biologically conceivable were included in multiple regressions. Statistical significance was assumed at *p <*0.05. All analyses were performed with SPSS (version 22.0, SPSS Inc., Chicago, IL, USA).

## Results

A total of 39 patients (11 men and 28 women) with a mean age of 50.28 ± 14.90 years were evaluated. Anthropometric and biochemical variables before and after correction of hypothyroidism are shown in **Table 1**. None of the patients had significant peripheral edema retention, ascites, or pleural effusion. The mean of the applied thyroxine dose for the correction of hypothyroidism was 139.74 ± 31.27 µg. The apelin level was 38% lower in the hypothyroid state than post-treatment, while copeptin levels did not change. Furthermore, serum sodium, chloride, potassium, estimated glomerular filtration rate (eGFR), NT-proBNP, adrenocorticotropic hormone (ACTH), and renin activity were significantly lower in the hypothyroid state. At the same time, BMI, abdominal circumference, and total body fat mass were higher during hypothyroidism. There was no difference in the extracellular fluid, intracellular fluid, serum osmolality, or urinary osmolality values. In the hypothyroid phase, hyponatremia was only detected in two patients (5.1%) in mild form and none of the euthyroid patients.

**Table 1:** Comparisons of selected anthropometric and biochemical characteristics between the two study phases.

Parameters	Hypothyroid phase	Control phase	*p*-value
Waist circumference (cm)	**94.13 ± 14.70**	**93.46 ± 14.50**	**0.039**
Body mass index	**29.10 (25.10–32.10)**	**28.20 (24.70–32.30)**	**<0.001**
Fat mass (kg)	**27.50 (21.40–32.30)**	**24.20 (18.00–31.90)**	**0.001**
Total body fluid (L)	38.30 (34.10–46.20)	37.80 (34.20–45.90)	0.624
Extracellular fluid (L)	16.80 (15.10–19.70)	17.30 (15.50–20.20)	0.673
Intracellular fluid (L)	22.30 (19.40–28.20)	22.40 (18.90–29.50)	0.451
TSH (mU/L)	**75.78 ± 25.28**	**2.45 ± 4.12**	**<0.001**
fT4 (pmol/L)	**2.93 ± 1.70**	**24.90 ± 5.71**	**<0.001**
Na (mmol/L)	**140.77 ± 2.60**	**142.33 ± 1.75**	**0.002**
Cl (mmol/L)	**100.82 ± 3.44**	**102.50 ± 2.63**	**0.012**
K (mmol/L)	**4.28 ± 0.35**	**4.41 ± 0.34**	**0.025**
eGFR (ml/min/1.73 m^2^)	**77.00 (61.00–90.00)**	**90.00 (72.00–90.00)**	**<0.001**
Uric acid (μmol/L)	295.28 ± 84.92	287.15 ± 91.77	0.363
Serum osmolality (mOsmol/kg)	287.49 ± 14.66	286.85 ± 7.14	0.817
Urine osmolality (mOsmol/kg)	530.79 ± 257.03	602.67 ± 253.90	0.136
Apelin	**1,901.88 ± 766.67 pg/ml = 1,226.39 ± 494.37 fmol/ml**	**3,080.65 ± 516.99 pg/ml = 1,986.49 ± 333.37 fmol/ml**	**<0.001**
Copeptin	1,007.7 (626.0–1,822.8) pg/ml = 243.7 (151.4–440.8) pmol/L	933.9 (619.6–1,437.4) g/ml = 225.8 (149.8–347.6) pmol/L	0.615
NT-proBNP (pg/ml)	**36.78 (20.59–73.73)**	**69.58 (37.37–194.92)**	**<0.001**
Renin activity (ng/ml/h)	**0.70 (0.42–1.63)**	**1.57 (0.92–2.85)**	**0.003**
Aldosterone (pg/ml)	166.50 (112.22–221.27)	174.23 (156.90–268.80)	0.18
ACTH (pg/ml)	**11.9 (10.00–16.00)**	**16.40 (12.20–22.50)**	**0.001**
Cortisol (nmol/L)	318.80 (242.30–385.90)	348.00 (275.60–440.20)	0.283

Significant differences are labeled as bold.

**Figure 1:**
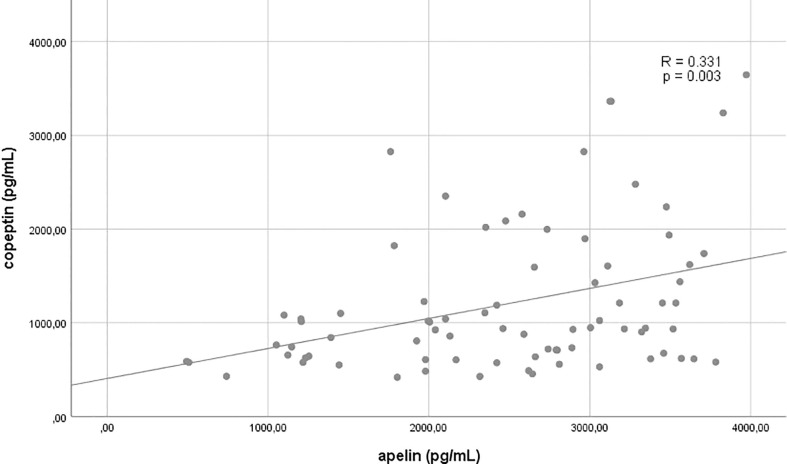
Correlation between copeptin and apelin.

**Figure 2:**
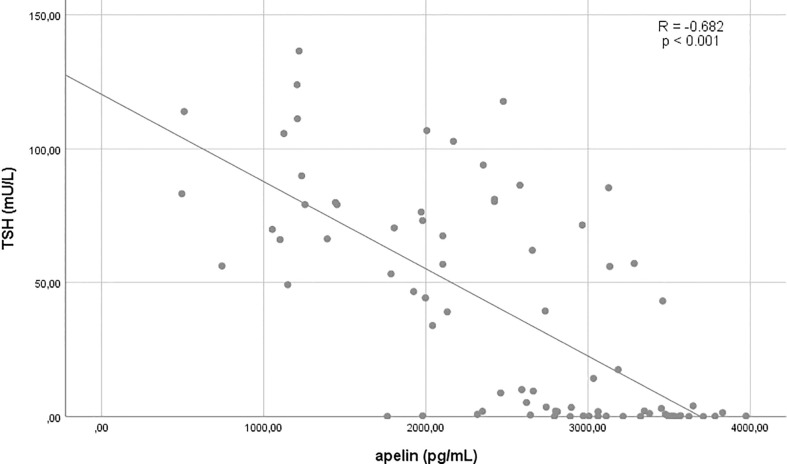
Correlation between TSH and apelin.

**Figure 3:**
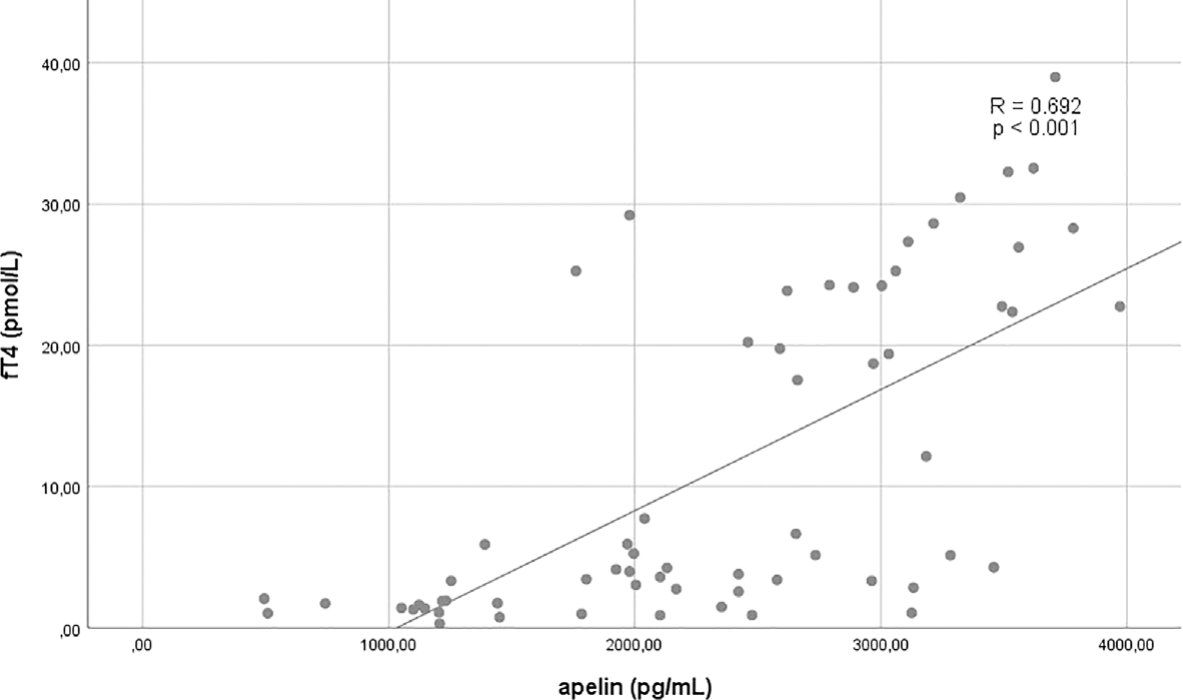
Correlation between fT4 and apelin.

The relations of apelin and copeptin to each other and selected other variables are shown in **Figures 1**–**3** and **Tables 2** **–** **5**. During the assessment of correlations, to broaden the investigated biochemical parameters, the whole database was used. Apelin and copeptin correlated with each other and serum sodium concentrations. Copeptin was not associated with any other investigated parameter, while apelin positively correlated with sodium, chloride, potassium, NT-proBNP, and fT4 and negatively correlated with TSH. Neither apelin nor copeptin was related to anthropometric variables, including BMI, fat mass, or body fluid values, and they did not differ among genders. In 10 patients, fT4 levels increased above the reference range due to the routinely taken thyroxine supplementation. None of the investigated parameters were different in this subpopulation compared to the rest of the patients (beyond TSH and fT4 levels). Regarding relevant humoral factors for fluid–ion homeostasis, changes in hypothyroidism and their correlations are summarized in **Table 3**.

**Table 2:** Relations of apelin and copeptin to the selected anthropometric and biochemical parameters.

Parameters	*R* values for apelin	*p*-value	*R* values for copeptin	*p*-value
Age	0.077	0.504	0.075	0.512
Waist circumference	0.004	0.969	0.035	0.761
Body mass index	−0.039	0.737	0.043	0.706
Fat mass	0.064	0.576	0.071	0.535
Total body fluid (L)	−0.198	0.082	0.096	0.403
Extracellular fluid (L)	−0.103	0.369	0.199	0.080
Intracellular fluid (L)	−0.173	0.130	0.063	0.586
TSH	**−0.682**	**<0.001**	−0.066	0.568
fT4	**0.692**	**<0.001**	0.067	0.599
Na	**0.349**	**0.002**	**0.226**	**0.046**
Cl	**0.451**	**<0.001**	0.186	0.115
K	**0.271**	**0.017**	0.176	0.122
eGFR	0.175	0.125	−0.131	0.251
Uric acid	−0.173	0.130	0.142	0.216
Serum osmolality (mOsmol/kg)	−0.031	0.789	0.155	0.177
Urine osmolality (mOsmol/kg)	0.091	0.426	−0.008	0.945
Apelin	1	1	**0.331**	**0.003**
NT-proBNP	**0.390**	**<0.001**	0.012	0.914
Renin activity	0.088	0.446	0.006	0.956
Aldosterone	0.097	0.402	0.113	0.326
ACTH	0.177	0.120	0.112	0.328
Cortisol	0.037	0.744	0.045	0.692

Significant associations are labeled as bold.

**Table 3:** Summary of relevant humoral factors for fluid–ion homeostasis; changes in hypothyroidism compared to the corresponding levels after thyroid hormone replacement and their correlations.

Humoral factor	Level in hypothyroidism	*R* values of correlations			
		Apelin	Copeptin	NT-proBNP	Aldosterone
Apelin	−**38%**	1	0.331**	0.390***	0.097
Copeptin	+7%	0.331**	1	0.012	0.113
NT-proBNP	−**48%**	0.390***	0.012	1	−0.087
Aldosterone	−5%	0.097	0.113	−0.087	1
Cortisol	−9%	0.037	0.045	−0.023	0.331**

Significant values are labeled as bold (**: p < 0.01, ***: p < 0.001).

Linear regression tests were run to test the potential independent predictors of either serum apelin or copeptin concentrations. The most representative results are shown in **Tables 4** and **5**. To widen the range of tested parameters during the regression analyses, they were carried out in the pooled data. Copeptin and TSH levels were significant determinants of serum apelin levels. TSH was especially strong, explaining almost half of the variance of the apelin concentrations. Regarding copeptin levels, aside from apelin, TSH was also an independent predictor, even though in a univariate test, no correlation was detected between them. However, TSH was a weaker determinant of copeptin concentrations than apelin (12.3% compared to 48.7%).

**Table 4:** A linear regression model with apelin as a dependent variable.

	Predictive power (%)	*p*-value
(Constant)		0.575
Age	0	0.869
Sex	1.5	0.114
BMI	1.4	0.441
Na	0.8	0.306
Copeptin	**13**	**<0.001**
TSH	**48.7**	**0.007**
fT4	1.3	0.184
NT-proBNP	0	0.890

Significant associations are labeled as bold.

**Table 5:** A linear regression model with copeptin as a dependent variable.

	Predictive power (%)	*p*-value
(Constant)		0.582
Age	0.5	0.530
Sex	0.1	0.835
BMI	2	0.146
Na	0	0.784
TSH	**12.3**	**0.001**
Apelin	**13.1**	**<0.001**

Significant associations are labeled as bold.

Serum sodium level had a variance of 5.47 mmol/L and, in addition to apelin and copeptin, was associated with the concentrations of TSH (*r* = −0.321; *p* = 0.004), ACTH (*r* = 0.349; *p* = 0.002), and cortisol (*r* = 0.225; *p* = 0.047). However, none of them were independent determinants of serum sodium levels in multiple regression analyses (data not shown).

## Discussion

Conflicting results were reported in the literature about apelin levels in hypothyroid patients. While some authors did not find a significant difference in subclinical or manifested hypothyroid patients (28, 33), others demonstrated lower apelin levels in subclinical hypothyroidism restored by levothyroxine supplementation (34). We can confirm these latter observations as we found significantly decreased apelin levels in our hypothyroid population. To the best of our knowledge, our investigation is the first one analyzing the relations of apelin to other humoral regulators of fluid–electrolyte homeostasis and the components of thyroid dysfunction. Of the investigated parameters, copeptin and TSH levels turned out to be predictors of serum apelin levels, with TSH being a much stronger determinant. In contrast, thyroxine levels did not have an impact.

In our study, hyponatremia was uncommon in hypothyroidism affecting only 5% of the patients. However, serum sodium concentrations were significantly lower in the hypothyroid phase, correlated with both apelin and copeptin levels in univariate analyses, but neither apelin nor copeptin levels were independent predictors of serum sodium, and vice versa, serum sodium was not an independent predictor of either apelin or copeptin levels. This might be explained by the relatively low variance of the serum sodium levels (i.e., 5.47 mmol/L) of our patients with free fluid intake compared to earlier studies investigating the effect of hypertonic saline infusion or water loading test (14). Interestingly, the alterations mentioned above were not present in the measured body fluid, serum, and urinary osmolality values.

Hyponatremia was rare and no alteration was detected in the ADH/AVP secretion (reflected by copeptin levels) of our patient population with short-term severe hypothyroidism. However, it can be hypothesized that a hypothyroidism-related decrease in apelin levels may contribute to the development of hyponatremia in some susceptible hypothyroid patients; namely, if simultaneous ADH/AVP oversecretion also evolves, for example as a side effect of a medication, due to high external temperature or increased fluid intake, hyponatremia may become manifested. The lower apelin levels may also contribute to the other comorbidities of hypothyroidism, e.g., accelerated atherosclerosis (31, 34).

There are conflicting data in the literature about ADH/AVP level changes in hypothyroid patients (4–7). This may be the consequence of the heterogeneity of the patient populations. Some authors only investigated young women with long-term hypothyroidism without any comorbidities (4). In chronic hypothyroidism, non-osmotic stimulation of ADH/AVP secretion by cardiac fibrosis and dysfunction may explain elevated serum ADH/AVP levels (4, 5). These alterations may be absent in short-term forms of hypothyroidism like in our model. In addition to the different patient populations, discordant results may be due to technical difficulties in measuring ADH/AVP levels. At the same time, copeptin is a more reliable surrogate marker of ADH/AVP (10). To the best of our knowledge, our study was the first to investigate the copeptin levels in hypothyroidism.

In our patients, copeptin and apelin had positive correlations with each other and the serum sodium level, contradictory to the expected reciprocal changes in copeptin and apelin plasma concentrations. Moreover, copeptin and apelin levels were significant independent determinants of each other’s serum concentrations during multiple regression analyses. Furthermore, TSH was also a predictor of both copeptin and apelin levels of which the association with apelin was especially strong, explaining almost half of its variance. Meanwhile, T4 was not a significant variable in these models. These findings may suggest that in this clinical setting the potential alterations of these hypothalamic hormones are not basically related to abnormalities in the osmotic or volemic state, but instead are regulated by hypothyroidism. Disturbances in the hypothalamic regulation may explain these findings, e.g., increased TRH secretion being present in hypothyroidism might be responsible for the low apelin level in this state.

Apelin or copeptin levels did not show associations with adrenal cortical hormones, and only the former was correlated to NT-proBNP concentrations. In line with previous observations of reduced ANH levels in the hypothyroid state (4, 32), we found significantly lower NT-proBNP concentrations. However, aldosterone concentrations were not reduced in our population, opposite to earlier findings in hypothyroid patients or healthy men challenged by hypertonic solution (4, 14). At the same time, renin activities were significantly elevated during the normalization of hypothyroidism, just like in previous studies (4, 35). The 89% elevation of NT-proBNP levels measured following the correction of hypothyroidism may at least partially explain the lack of significant changes in aldosterone concentrations. The aldosterone secretion stimulating effects of the increased renin activity and potassium concentrations may be counterbalanced by the simultaneous elevation of ANH levels (measured as NT-proBNP) during the correction of hypothyroidism. Moreover, the increase in apelin levels following the correction of hypothyroidism may contribute to the elevation of NT-proBNP due to its hemodynamic effects.

Understanding the regulation of apelin secretion is key as it plays a pivotal role in obesity, diabetes, cancer, heart failure, increasing cardiac output, and hypoxia-related diseases attenuating oxidative stress. Moreover, this peptide can be treated as a biomarker for cardiovascular diseases and a protector against apoptosis (31).

The greatest advantage of our study is that multiple potentially interrelated humoral factors were evaluated in a homogeneous patient population without comorbidities that could distort the obtained results. We attempted to understand the potential pathomechanisms of our basic observations by applying various statistical methods. However, this approach has inherent limitations, and therefore, multiple findings cannot be reliably explained. Another shortcoming of our study is the lack of a normal control population. However, matching controls in multiple parameters beyond usual anthropometric ones would have been difficult, and the self-control pattern of our study allowed us to achieve more or less standardized experimental conditions. Furthermore, our population consisted of relatively few participants. However, many of our original observations and relationships were statistically highly significant.

## Data availability statement

The raw data supporting the conclusions of this article will be made available by the authors, without undue reservation.

## Ethics statement

The studies involving human participants were reviewed and approved by Ethics Committee at the Medical Center of the University of Pécs (6961/2017). The patients/participants provided their written informed consent to participate in this study.

## Author contributions

MG, EM, and LB designed the study, analyzed the data, and wrote the manuscript. MG measured the body composition and conducted the statistical analysis. GP-D participated in the evaluation of laboratory results. ZH-S carried out the ELISA tests. TK supervised the laboratory investigations. All authors contributed to the article and approved the submitted version.

## Funding

The research was financed by the Higher Education Institutional Excellence Program of the Ministry of Human Capacities in Hungary, within the framework of the 2. thematic program of the University of Pécs.

## Acknowledgments

We would like to thank Erzsébet Györgyi for her valuable and constructive assistance during the laboratory measurements and Anna Bajnok for her assistance in improving the language of the manuscript.

## Conflict of interest

The authors declare that the research was conducted in the absence of any commercial or financial relationships that could be construed as a potential conflict of interest.

## Publisher’s note

All claims expressed in this article are solely those of the authors and do not necessarily represent those of their affiliated organizations, or those of the publisher, the editors and the reviewers. Any product that may be evaluated in this article, or claim that may be made by its manufacturer, is not guaranteed or endorsed by the publisher.

## Abbreviations

ACTH, adrenocorticotropic hormone; ADH, antidiuretic hormone; ANH, atrial natriuretic hormone; AVP, arginine vasopressin; BIA, bioelectrical impedance analysis; BMI, body mass index; DTC, differentiated thyroid cancer; eGFR, estimated glomerular filtration rate; fT3, free triiodothyronine; fT4, free thyroxin; GFR, glomerular filtration rate; NT-proBNP, N-terminal prohormone of brain natriuretic peptide; RAI, radioiodine; RAS, renin–angiotensin system; SIAD, syndrome of inappropriate antidiuretic hormone secretion; TRH, thyrotropin-releasing hormone; TSH, thyroid-stimulating hormone.
